# Mitochondrial alterations in fibroblasts from sporadic Alzheimer's disease (AD) patients correlate with AD-related clinical hallmarks

**DOI:** 10.1186/s40478-024-01807-x

**Published:** 2024-06-08

**Authors:** Fanny Eysert, Paula-Fernanda Kinoshita, Julien Lagarde, Sandra Lacas-Gervais, Laura Xicota, Guillaume Dorothée, Michel Bottlaender, Frédéric Checler, Marie-Claude Potier, Marie Sarazin, Mounia Chami

**Affiliations:** 1 INSERM, CNRS, Institute of Molecular and Cellular Pharmacology, Laboratory of Excellence DistALZ, Université Côte d’Azur, 660 Route des Lucioles, 06560 Sophia-Antipolis, Valbonne, France; 2 Department of Neurology of Memory and Language, GHU Paris Psychiatrie & Neurosciences, Hôpital Sainte Anne, 75014 Paris, France; 3 Université Paris-Cité, 75006 Paris, France; 4 BioMaps, Service Hospitalier Frédéric Joliot CEA, CNRS, Inserm, Université Paris-Saclay, 91401 Orsay, France; 5 Centre Commun de Microscopie Appliquée, Université de Nice Côte d’Azur, 06108 Nice, France; 6 UPMC University Paris 06, UMRS 1127, Sorbonne Universités, Paris, France; 7 ICM Research Center, CNRS UMR 7225, Paris, France; 8 Inserm, Centre de Recherche Saint-Antoine, CRSA, Immune System and Neuroinflammation Laboratory, Hôpital Saint-Antoine, Sorbonne Université, 75012 Paris, France; 9 UNIACT, Neurospin, Joliot Institute, CEA, Université Paris-Saclay, 91140 Gif sur Yvette, France

**Keywords:** Alzheimer’s disease, Mitochondria, Mitophagy, Biomarker, Fibroblasts, Clinical correlations

## Abstract

Mitochondrial dysfunctions are key features of Alzheimer’s disease (AD). The occurrence of these disturbances in the peripheral cells of AD patients and their potential correlation with disease progression are underinvestigated. We studied mitochondrial structure, function and mitophagy in fibroblasts from healthy volunteers and AD patients at the prodromal (AD-MCI) or demented (AD-D) stages. We carried out correlation studies with clinical cognitive scores, namely, (i) Mini-Mental State Examination (MMSE) and (ii) Dementia Rating-Scale Sum of Boxes (CDR-SOB), and with (iii) amyloid beta (Aβ) plaque burden (PiB-PET imaging) and (iv) the accumulation of peripheral amyloid precursor protein C-terminal fragments (APP-CTFs). We revealed alterations in mitochondrial structure as well as specific mitochondrial dysfunction signatures in AD-MCI and AD-D fibroblasts and revealed that defective mitophagy and autophagy are linked to impaired lysosomal activity in AD-D fibroblasts. We reported significant correlations of a subset of these dysfunctions with cognitive decline, AD-related clinical hallmarks and peripheral APP-CTFs accumulation. This study emphasizes the potential use of peripheral cells for investigating AD pathophysiology.

**Supplementary Information:**

The online version contains supplementary material available at 10.1186/s40478-024-01807-x.

## Introduction

Studies on mitochondria in the field of Alzheimer’s disease (AD) identified novel pathophysiological mechanisms accounting for disease development. It is now recognized that mitochondrial dysfunctions contribute to the neurodegeneration that occurs in AD [1, 2]. Indeed, mitochondrial dysfunctions have been observed in postmortem human brain samples and were demonstrated in AD cellular models as well as in the brains of AD mice [1]. These alterations include impaired mitochondrial membrane potential (ΔΨm), reduced ATP production, increased mitochondrial reactive oxygen species (mitROS) levels and altered mitochondrial fission and fusion balance [3, 4]. Moreover, mitophagy, a specific process that removes damaged mitochondria, is altered in AD [5, 6]. The accumulation of damaged mitochondria is a source of noxious material (e.g., toxic mitROS), likely contributing to a vicious cycle driving disease progression.

In the brains of transgenic AD mice, mitochondrial dysfunctions and mitophagy defects occur early since they are observed before the appearance of extracellular deposits of β-amyloid peptide (Aβ) in senile plaques [3]. These Aβ peptides are among the products of the cleavage of the transmembrane amyloid precursor protein (APP). In the amyloidogenic pathway, APP is first cleaved by β-secretase, generating an APP C-terminal fragment (APP-CTF) of 99 amino acids (C99) that is further cleaved by γ-secretase to produce Aβ peptides and the amyloid precursor protein intracellular domain (AICD). Alternatively, APP and C99 can also be cleaved by α-secretase, producing an APP-CTF, which is composed of 83 amino acids (C83) [7]. We recently demonstrated that the accumulation of APP-CTFs (C99 and C83) in cellular models triggers, in an Aβ-independent manner, excessive alterations in mitochondrial morphology as well as enhanced mitROS production and mitophagy impairment [5]. We further demonstrated alterations in mitochondrial structure and mitophagy in the brains of transgenic mice in which APP-CTFs accumulated and confirmed these findings in human postmortem sporadic AD brains [5]. Accordingly, defects in general autophagy and lysosomal activity in AD have been associated with APP-CTFs accumulation [8–10].

Mitochondrial dysfunctions in AD are not restricted to the brain. Indeed, several studies have reported alterations in mitochondrial structure and function in peripheral cells from AD patients, such as lymphocytes, peripheral blood mononuclear cells (PBMCs) and fibroblasts [11–13]. Other studies have revealed that mitochondrial dysfunctions occur in peripheral cells of familial forms of the disease (FAD) [14, 15], representing 1% of AD cases, and are linked to mutations in APP or presenilin 1 and 2 genes (coding for the catalytic core of γ-secretase), as well as in sporadic AD (SAD) [12], with non-Mendelian (complex) transmission and representing the majority of AD cases [16]. However, it remains uncertain whether these mitochondrial dysfunctions in peripheral AD cells occur at the prodromal stage and could thus be considered potential early hallmarks correlated with clinical severity or pathophysiological AD markers.

We studied fibroblasts obtained from a well-characterized cohort of healthy volunteers and SAD patients at mild cognitive impairment (AD-MCI) or dementia (AD-D) stages [17, 18] and investigated several aspects of mitochondrial structure and function as well as the key steps underlying mitophagy and autophagy. The originality of the current study also stands in the correlative analyses between the observed alterations and the clinical data.

## Materials and methods

### Patients and control individuals

Human primary fibroblasts were obtained from the IMABio3 and Shatau7-Imatau study cohorts (NCT01775696 and NCT02576821-EudraCT2015-000257-20) (CTRL, n = 9; AD-MCI, n = 11; AD-D, n = 9), which were approved by the French Ethics Committee. All the subjects provided written informed consent prior to participating.

Patients with AD were included according to the following criteria: (i) cognitive impairment characterized by a predominant progressive episodic memory deficit and (ii) positive pathophysiological markers of AD, defined by CSF AD profile and amyloid Pittsburgh compound B (PiB)-PET imaging. Controls were recruited according to the following criteria: (i) Mini-Mental State Examination (MMSE) score ≥ 27/30 and normal neuropsychological assessment; (ii) Clinical Dementia Rating-Scale Sum of Boxes (CDR-SOB) equal to 0; (iii) no history of neurological or psychiatric disorders; (iv) no memory complaint or cognitive deficit; and (v) amyloid PiB-PET imaging < 1.5 except for one individual who had a PiB-GCI value of 2.18 but a CDR-SOB equal to 0 and an MMSE score of 30 at the inclusion date and who was clinically stable over two years after inclusion. We did not include subjects with severe cortical or subcortical vascular lesions, a history of autoimmune and inflammatory diseases or psychiatric disorders or suspected alcohol or drug abuse. Participants followed a complete clinical and neuropsychological assessment and ^11^C-PiB PET imaging as previously described [18, 19] and were followed up annually for 2 years. Skin biopsies were obtained during the 1-year or final (2-year) visit. The demographic characteristics of the patients according to diagnostic group are described in Table 1.

**Table 1:** Demographic characteristics of the IMABio3 cohort

Group	# of patients	Age	Gender	MMSE	CDR-SOB	PiB-GCI
CTRL	9	51–81	4F/5 M	29.3 ± 0.86	0	1.43 ± 0.30
AD-MCI	11	52–79	5F/6 M	24.3 ± 2.97	2.50 ± 1.07	2.75 ± 0.57
AD-D	9	51–81	8F/1 M	16.5 ± 3.36	7.21 ± 0.69	3.00 ± 0.54

The CDR-SOB (Clinical Dementia Rating, Scale Sum of Boxes) scale indicates the severity of dementia; no dementia (CDR = 0). The maximum score for the Mini-Mental State Examination (MMSE) is 30, with lower scores associated with greater cognitive deterioration

*CTRL* control; *AD-MCI* Alzheimer’s disease patients with mild-cognitive impairment; *AD-D* Alzheimer’s disease patients with dementia; *F* female; *M* male; *CDR-SOB* clinical dementia rating sum of the boxes; *PiB-GCI* ^11^C‐labelled Pittsburgh compound B, Global cortical index

### Primary fibroblast culture

Fibroblasts were cultured as described previously [20] and kept under liquid nitrogen at the DNA & Cell Bank core facilities of the Brain and Spinal Cord Institute (ICM). Briefly, cells were cultured at 37 °C in a humidified atmosphere containing 5% CO_2_ in Dulbecco’s modified Eagle's minimal essential medium (DMEM) supplemented with 10% heat-inactivated fetal bovine serum (FBS), 1% pyruvate sodium, penicillin (100 U/mL) and streptomycin (50 μg/mL). All experiments were performed using cells between the 4th and 12th passages. The experiments were performed on batches from the same passages.

### Chemicals and antibodies

When indicated, fibroblasts were treated with the respiratory chain uncoupling agent carbonyl cyanide 3-chlorophenylhydrazone (CCCP) at 10 µM (Millipore-Sigma, C2759) for 6 h.

We used the following commercially available antibodies: β-actin (A5316, Sigma‒Aldrich), APP-Cter (A8717, Gift from P. Fraser, Toronto), Chaperonin 10 (CPN10) (ADI-SPA-110, Enzo Life Sciences), CoxII (12C4F12, Thermo Fisher Scientific), Parkin (MAB5512, Millipore) and TOMM20 (612278, BD Transduction Laboratories). The secondary antibodies used for western blot studies were goat anti-mouse and goat anti-rabbit (115-036-003 and 111-036-045, respectively, Jackson ImmunoResearch).

### Mitochondrial fraction preparation and SDS‒PAGE analysis

Cells were harvested with 0.05% trypsin (Gibco), washed with PBS, sedimented by centrifugation and resuspended in isolation buffer (250 mM D-mannitol, 5 mM HEPES pH 7.4, 0.5 mM EGTA, and 0.1% BSA) supplemented with a protease inhibitor mixture (Sigma, P2714-1BTL). After 15 min of incubation on ice, the cells were disrupted by 100 strokes of a glass Dounce homogenizer. The lysates were centrifuged at 600 × *g* at 4 °C for 5 min to remove unbroken cells and nuclei. The supernatant was centrifuged at 10,000 × *g* at 4 °C for 10 min to pellet the mitochondrial fraction, which was subsequently suspended in isolation buffer supplemented with protease inhibitors. The protein concentration was determined according to the Bradford method (Bio-Rad protein Assay, Spectrophotemeter Eppendorf biophotometer plus at 595 nm). Full-length APP and APP-CTFs were resolved by 16.5% Tris-Tricine SDS‒PAGE and then transferred onto nitrocellulose membranes that were boiled in PBS. All the other proteins were resolved by 16% Tris-glycine SDS‒PAGE. Then, the membranes were saturated in 5% skim milk TBS tween buffer (TBS-T) and incubated overnight at 4 °C with specific primary antibodies. After washing with TBS-T, the membranes were incubated with HRP-conjugated antibodies (Jackson ImmunoResearch, 1/5000) for 1 h at room temperature. The membranes were rinsed with TBS-T and visualized using an ImageQuant LAS4000.

### Confocal imaging analyses

Twenty-four hours after seeding on glass coverslips, the cells were transiently transfected with LC3-GFP (Addgene plasmid #11546), the LAMP1-GFP probe (Addgene plasmid #34831) [21] and the mitochondrial Mit-RFP probe [22] using jetPRIME (polyplus transfection, #114-15) according to the manufacturer’s instructions.

Cells transfected with the Mit-RFP probe were treated for 24 h with drugs as indicated and fixed with 4% paraformaldehyde for 15 min at room temperature. Other cells were incubated for 30 min with LysoTracker Red DND-99 (1:20,000, Invitrogen) at 37 °C and fixed with 4% PFA. The cells were rinsed with PBS and stained with DAPI (1:10,000, Roche) before being coverslipped on glass slides with Vectamount medium (Vector). The fixed cells were visualized using a Leica SP5 microscope with a 63X objective. Colocalization was quantified using the Fiji plug-in JACoP (Just Another Colocalization Plug-in) [23]. LC3 and LAMP1 dot sizes and counts and LysoTracker intensity were quantified using the Analyze Particles function of Fiji software. The mitochondrial network volume was quantified using the 3D object counter plugin of Fiji.

We also analyzed the structure of the mitochondrial network skeleton, including the mitochondrial branch length and junction number, using the skeletonize plugin Fiji [24]. The quantification was performed randomly in and based only on the presence of a mitochondrial red signal representing cells transfected with the mit-RFP probe or cotransfected with mit-RFP and LC3-GFP or LAMP1-GFP.

### In vitro cathepsin D activity assay

CTRL, AD-MCI and AD-D fibroblasts were harvested with 5 mM PBS-EDTA, centrifuged at 600 × *g* for 5 min, washed in PBS and centrifuged again at 600 × *g* for 5 min. The pellet was subsequently resuspended in Tris-HCl (10 mM, pH 7.5). To monitor cathepsin D (CTSD) activity, 25 μg of protein extracts were incubated in acetate buffer (25 mM, pH 4.5, and 8 mM L-cysteine HCl) containing CTSD substrate (Enzo BML-P145-001, 50 µM) in the absence or presence of pepstatin A (20 μM, Sigma). CTSD activity corresponds to the pepstatin A-sensitive fluorescence recorded at 320 nm (excitation) and 420 nm (emission) using a fluorescence plate reader (Varioskan, Thermo Fisher Scientific). The fluorescence was recorded every 30 s for 45 min, and the CTSD activity was calculated as the slope in the linear range corresponding to the initial 5 min.

### Mitochondrial potential and mitochondrial superoxide measurements

Tetramethyl rhodamine methyl ester (TMRM) (Thermo Fisher, #T668) is a cell-permanent dye that accumulates in active mitochondria with intact membrane potentials. The loss of mitochondrial membrane potential (depolarization) is reflected by reduced TMRM accumulation. In contrast, an increase in the mitochondrial membrane potential (hyperpolarization) is reflected by elevated TMRM intensity [25]. MitoSOX Red (Invitrogen, M36008) is a fluorogenic dye for highly selective detection of superoxide in the mitochondria of living cells [26]. The cells were rinsed with PBS and incubated with TMRM at 2 nM or MitoSOX at 5 µM in DMEM for 30 min at 37 °C. Cells stained with the MitoSOX probe were rinsed before acquisition with HBSS-CaCl_2_-MgCl_2_ (1 mM CaCl_2_, 0.5 mM MgCl_2,_ and HBSS). The TMRM and MitoSOX fluorescence intensities were analyzed by flux cytometry.

### Seahorse Mito stress test and ATP rate analyses

Measurements of aerobic respiration and glycolysis were conducted with a Seahorse Bioscience XFe96 bioanalyzer using a Seahorse XF Mito Stress Test Kit (Agilent #103015-100) and a Seahorse XF Real-Time ATP Rate Assay Kit (Agilent #103592-100). Fifteen thousand cells per well were seeded on XFe96 cell culture microplates (Agilent #102416-100) one day before the experiment. On the day of the experiment, the culture medium was replaced with Seahorse Base Medium (Agilent #103334-100) supplemented with 1 mM pyruvate, 2 mM glutamine and 10 mM glucose, and the cells were incubated for 30 min at 37 °C in a CO_2_-free incubator. For the Seahorse XF Real-Time ATP Rate Assay, the medium was replaced a second time after the incubation, before the cell culture microplate was loaded into the Seahorse Analyzer. After measuring basal respiration, oligomycin (5 µM), FCCP (2 µM), and rotenone/antimycin A (0.5 µM each) were added in a sequential order to each well for the XF Mito Stress Test, while oligomycin and rotenone/antimycin A were added for the Real-Time ATP Rate Assay. The data were analyzed using the XF Cell Mito Stress Test or the XF Cell Real-Time ATP Rate Report Generator. After the assay, fibroblasts were fixed with 4% PFA and stained with DAPI (Roche; 1:10,000) for 5 min. A Cytation 5 Biotek was then used to count the number of cell nuclei (cell number) in each well. The normalization of the data was based on the relative cell number per well.

### Transmission *electron* microscopy (TEM)

For mitochondrial ultrastructure analysis, cells were fixed in 1.6% glutaraldehyde in 0.1 M phosphate buffer (pH 7.4), rinsed with 0.1 M cacodylate buffer, and then postfixed in osmium tetroxide (1% in cacodylate buffer) reduced with potassium ferrycyanide (1%) for 1 h. The cells were dehydrated with several incubations in increasing concentrations of ethanol or acetone and embedded in epoxy resin (EPON), and 70 nm ultrathin sections were contrasted with uranyl acetate and lead citrate and observed with a transmission electron microscope (JEOL JEM 1400) operating at 100 kV and equipped with an Olympus SIS MORADA camera. Images were obtained in a blinded manner, and Fiji software was used to analyze the mitochondrial ultrastructure. The quantification of mitochondrial classes was first performed from individual cells from the same patient, and the means ± SEMs of each mitochondrial class were then reported for each fibroblast group.

### Statistical analyses

The data are expressed as the means ± SEMs. The sample size for each experiment is indicated in the Figure captions. The data were analyzed with GraphPad Prism version 9 for Windows (GraphPad Software, La Jolla, CA, USA; https://www.graphpad.com). The data were first analyzed for a normal distribution. We used one-way ANOVA with Dunnett’s or Tukey’s multiple comparisons post hoc test when groups of two or more variables passed the normality test. The Kruskal–Wallis test and Dunn’s multiple comparisons post hoc test were used when groups of variables did not pass the normality test. Correlation analyses were performed by linear regression to determine* P* and goodness of fit (R^2^) values taking into consideration the age of patients as well as the delay between the date of patient inclusion and the date of skin biopsy as covariates using InVivoStat software. Significant differences are as follows: *p < 0.05, **p < 0 0.01, ***p < 0.001, ****p < 0.0001 and ns: not significant.

## Results

### Mitochondrial ultrastructure is altered in AD-D fibroblasts and is associated with cognitive impairments

**Fig. 1:**
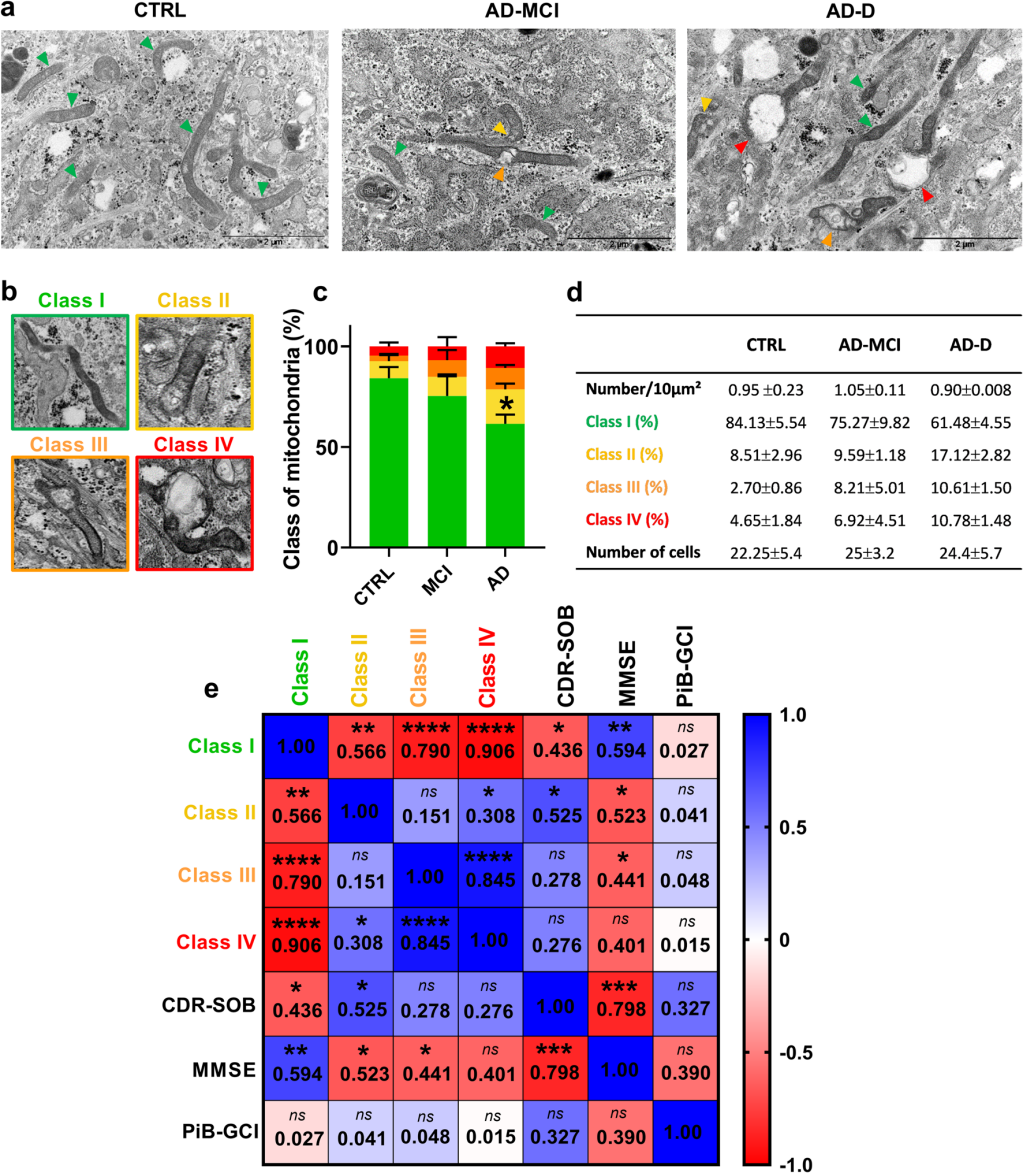
Mitochondrial classes distribution differ among CTRL, AD-MCI and AD-D fibroblasts and correlates with cognitive impairments. **a** Representative electron microscopy images of the ultrastructures of CTRL, AD-MCI and AD-D fibroblasts. N: nuclei. The colored arrowheads indicate mitochondria (green = class I, yellow = class II, orange = class III and red = class IV). Scale bar = 2 µm. **b** Representative images of mitochondrial classes I, II, III, and IV. **c** Quantitative graph of the distribution of mitochondrial classes ± SEM. * *p* = 0.013 according to 2-way ANOVA and Tukey’s multiple comparisons test versus CTRL class I mitochondria. **d** Table presenting the average number ± SEM of mitochondria per 10 µm^2^ and the percentage of mitochondria classes ± SEM obtained from CTRL (n = 4), AD-MCI (n = 4) and AD-D (n = 5) fibroblasts. Mitochondrial ultrastructure was quantified in the CTRL group (4 individuals, n = 89 cells), AD-MCI group (4 patients, n = 100 cells) and AD-D group (4 patients, n = 122 cells) (between 100 and 300 mitochondria/patient). **e** Heatmap of the correlation matrix computed linear regression value (R^2^) between mitochondrial classes (I, II, III and IV) and the clinical Dementia Rating (Sum of Boxes) (CDR-SOB), the Mini Mental State Examination (MMSE) and the β-amyloid plaque burden assessed using the ^11^C‐labelled Pittsburgh compound B (Global Cortical Index) (PiB-GCI) through positron emission tomography (PET-scan) at patient inclusion. A color scale of 1 corresponds to the maximum positive correlation value (blue), and a scale of − 1 corresponds to the maximum negative correlation value (red). *p < 0.05; **p < 0.01; ***p < 0.001; ****p < 0.0001 for the correlation of every pair of data sets

We assessed mitochondrial ultrastructure by transmission electron microscopy (TEM) and revealed that the number of mitochondria did not differ among fibroblasts isolated from CTRL individuals, AD-MCI patients and AD-D patients (Fig. 1a, d). We further examined mitochondrial morphology and size and classified mitochondrial ultrastructures into four classes based on the organization of the cristae and the density of the matrix as previously described [5] (class I: healthy dark mitochondria with a uniform matrix filled with dense regularly distributed cristae; class II: mitochondria with disrupted cristae and loss of matrix density; class III: empty mitochondria with disorganized cristae; and class IV: swollen mitochondria with disorganized cristae) (Fig. 1a, b). This classification revealed a majority of healthy class I mitochondria (84.13%) and a minor proportion of class II (8.51%), class III (2.70%) and class IV (4.65%) mitochondria in CTRL fibroblasts (Fig. 1c, d). We then reported that the distribution of mitochondrial subtypes is modified in AD fibroblasts. Indeed, AD-MCI and AD-D fibroblasts displayed fewer healthy class I mitochondria (75.27% and 61.48%, respectively) and more class II (9.59% and 17.12%, respectively), class III (8.21% and 10.61%, respectively) and class IV (6.92% and 10.78%, respectively) mitochondria than did CTRL cells (Fig. 1c, d). Importantly, the percentage of mitochondria class I in AD-D fibroblasts was significantly lower than that in CTRL fibroblasts.

The correlation analyses, which included CTRL, AD-MCI and AD-D fibroblasts, integrated mitochondrial classes, CDR-SOB and MMSE scores and PiB-GCI data and considered the age at patient inclusion as well as the delay between the date of inclusion and the date of skin biopsy as covariates, revealed, as expected, (i) significant negative correlations between healthy (class I) and damaged mitochondria (classes II, III, and IV) (Fig. 1e) and (ii) a significant inverse correlation between the CDR-SOB score and the MMSE score (Fig. 1e, and Table 1). Importantly, we demonstrated that the MMSE score was significantly positively correlated with the number of healthy mitochondria (class I) and significantly negatively correlated with the number of damaged mitochondria (class II, III and IV) (Fig. 1e). In parallel, we reported that the CDR-SOB score was significantly negatively correlated with the number of healthy mitochondria (class I) and was significantly positively correlated with the number of dysfunctional mitochondria (mitochondria class II) (Fig. 1e). However, these alterations in the mitochondrial ultrastructure did not correlate with the levels of PiB-GCI (Fig. 1e), tau, p-tau, or Aβ42 in the CSF (data not shown).

Taken together, these data demonstrate a relevant alteration in mitochondrial ultrastructure in AD-D versus CTRL fibroblasts. These alterations in the mitochondrial ultrastructure are closely associated with cognitive impairment (CDR-SOB and MMSE scores).

### Mitochondrial volume and network organization are impacted in AD-D fibroblasts

**Fig. 2:**
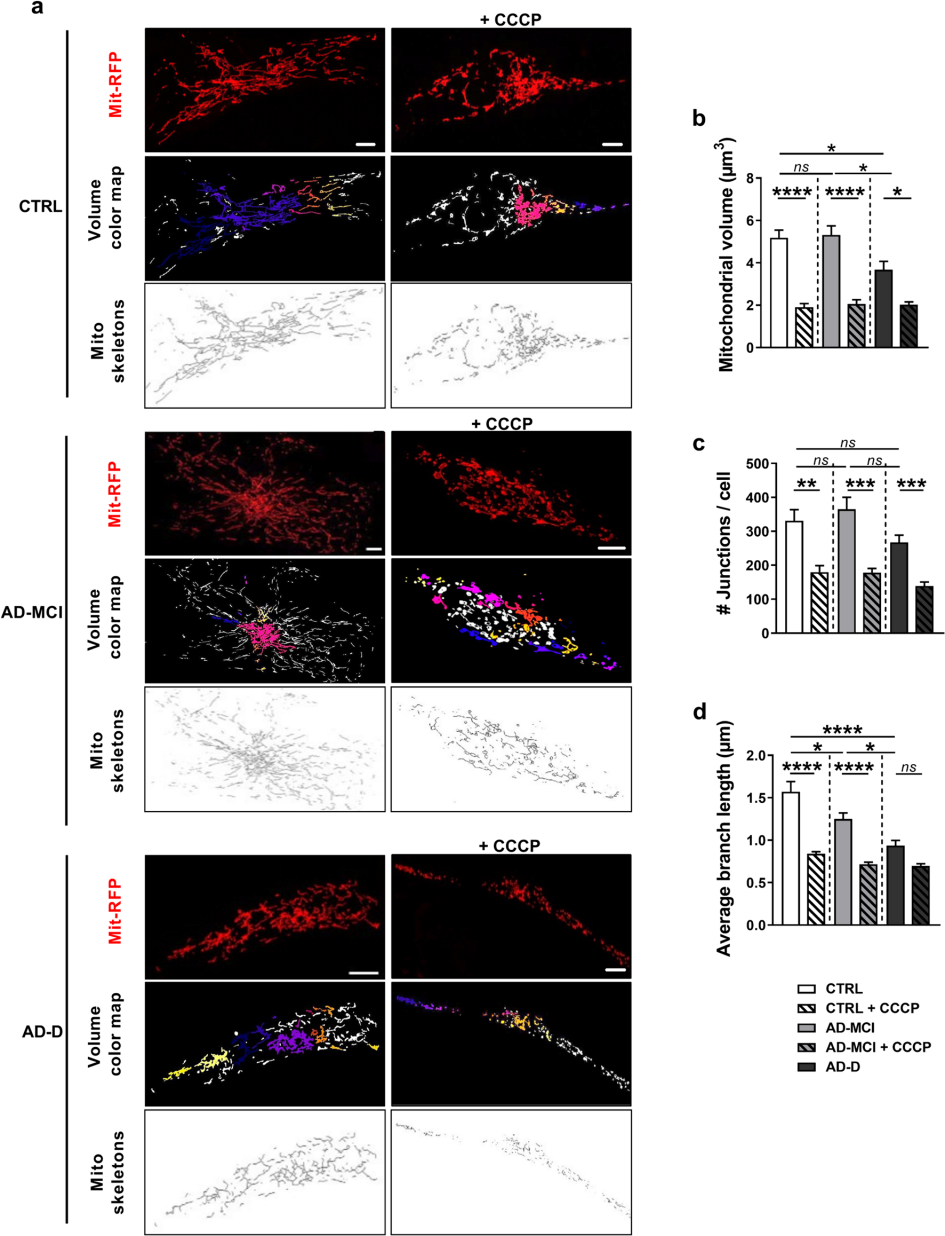
Mitochondrial volume and network organization are more strongly impacted in AD-D fibroblasts than in AD-MCI fibroblasts. **a** Representative images of mitochondrial network morphology in CTRL, AD-MCI and AD-D fibroblasts transfected with the Mit-RFP probe and treated with or without 10 µM CCCP for 6 h. Scale bar = 2 µm. The original images were processed with the Fiji 3D Counter or skeletonize plugin providing the volume color map and the mitochondrial network skeletons. The mitochondrial volume, number of junctions and branch length were quantified in the CTRL group (4 individuals, n = 59 cells), AD-MCI group (4 patients, n = 61 cells) and AD-D group (4 patients, n = 56 cells). **b**–**d** Quantitative graphs of the mitochondrial volume (µm^3^) (**b**), the number of junctions (**c**) and the mean branch length (µm) (**d**) in the mitochondrial network in individual cells. Means ± SEM were obtained for the CTRL (n = 4), AD-MCI (n = 4) and AD-D (n = 4) groups. *p < 0.05; **p < 0.01; ***p < 0.001; ****p < 0.0001

To further consolidate the results obtained by TEM, we analyzed in depth mitochondrial morphology using confocal imaging and three-dimensional (3D) reconstitution of the mitochondrial network in fibroblasts transiently transfected with the Mit-RFP probe (Fig. 2a). To investigate the potential differences between AD-MCI, AD-D and CTRL fibroblasts under mitochondrial stress conditions, these analyses were performed under basal conditions and upon CCCP treatment, which is known to trigger mitochondrial membrane depolarization and mitochondrial network fragmentation. As expected, CCCP treatment decreased the mitochondrial volume in the three groups (Fig. 2b). Interestingly, we reported a significant decrease in the mitochondrial volume in AD-D fibroblasts compared to that in CTRL and AD-MCI cells under basal conditions. These results suggest a trend toward fragmentation of the mitochondrial network in AD-D fibroblasts compared to that in AD-MCI and CTRL cells.

To fully test this hypothesis, we evaluated several mitochondrial structure features, including the average number of junctions (Fig. 2c) and the average branch length in the mitochondrial network per single cell (Fig. 2d). Consistent with the results of the mitochondrial volume analyses (Fig. 2b), our data revealed no significant differences in the number of junctions between AD-MCI and CTRL fibroblasts under basal conditions (Fig. 2c) and revealed a decreasing trend in these parameters in AD-D fibroblasts (Fig. 2c). Specifically, CCCP treatment significantly reduced the number of junctions in the three groups of cells (Fig. 2c). Importantly, we noticed a significant decrease in the average branch length of the mitochondrial network in AD-MCI and AD-D cells compared to that in CTRL cells (Fig. 2d) and between AD-MCI and AD-D cells (Fig. 2d). In addition, CCCP treatment drastically reduced the average branch length in CTRL and AD-MCI fibroblasts to that observed in AD-D fibroblasts (Fig. 2d). Together, these analyses demonstrated a progressive alteration in the mitochondrial network in AD-D fibroblasts compared with that in AD-MCI and CTRL fibroblasts.

### Mitochondrial function and related metabolic activities are dysfunctional in AD-MCI and AD-D fibroblasts

**Fig. 3:**
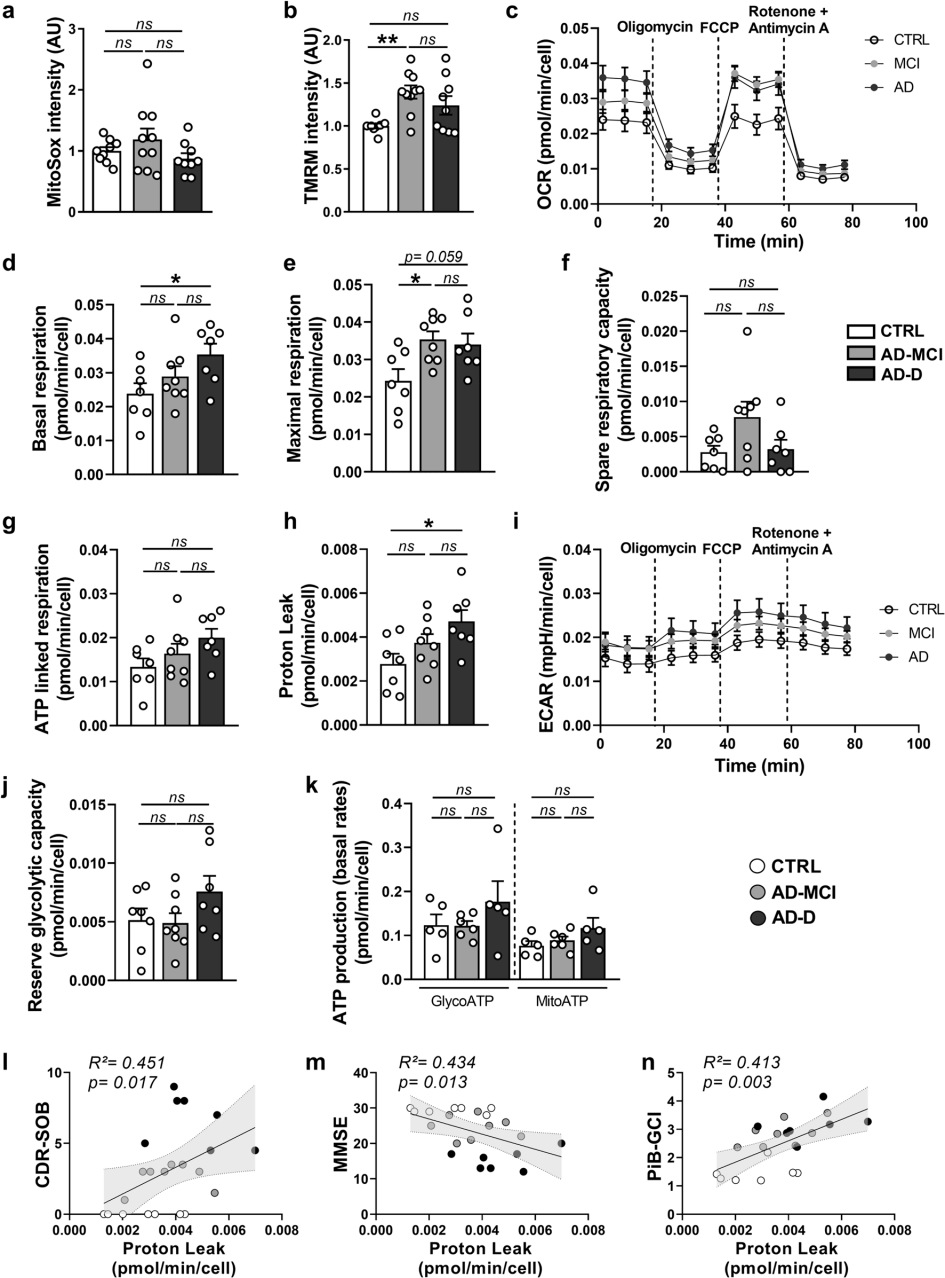
Mitochondrial function in AD-D fibroblasts is altered in a disease-specific manner in AD-MCI patients. **a** Quantitative graph of MitoSOX mean intensity ± SEM obtained by flux cytometry analyses of the CTRL (n = 9), AD-MCI (n = 10) and AD-D (n = 9) groups. ns: not significant. **b** Quantitative graph of TMRM mean intensity ± SEM obtained by flux cytometry analyses of the CTRL (n = 8), AD-MCI (n = 10) and AD-D (n = 9) groups. **p < 0.01; ns: not significant. **c**–**j** Cell respiratory capacity in CTRL (n = 7), AD-MCI (n = 8) and AD-D (n = 7) fibroblasts determined using a SeaHorse XFe96 extracellular flux analyzer (Mito Stress Test). **k** ATP production measured using a SeaHorse XFe96 extracellular flux analyzer (ATP rate assay) in CTRL (n = 5), AD-MCI (n = 6) and AD-D (n = 5) fibroblasts. The data are expressed in pmol/min/cell ± SEM or mpH/min/cell ± SEM obtained from 5 replicates for each type of fibroblast. *p < 0.05; **p < 0.01; ****p < 0.0001; ns: not significant. **l**–**n** Correlation plots between proton leakage and the CDR-SOB (**l**), MMSE (**m**), and PiB-GCI (**n**) scores at patient inclusion, including the CTRL (white dots), AD-MCI (gray dots) and AD-D (black dots) groups. Linear regression was used to determine *the P* and goodness of fit (R^2^) values

As mitochondrial structure is altered in AD-MCI and AD-D fibroblasts, we hypothesized that mitochondrial function could also be impaired. First, we assessed mitochondrial ROS (mitROS) levels by using a MitoSOX probe and flux cytometry analyses and reported no differences in mitROS production between groups of fibroblasts (Fig. 3a). Since the obtained results were homogeneous inside each group, we then assessed mitROS production under stress conditions triggered by oligomycin and antimycin A (OA) (known to block mitochondrial ATPase and respiratory chain complex III, respectively) in a set of fibroblasts using the MitoSOX probe and confocal microscopy analyses (Fig. S1a, b). The MitoSOX intensity in AD-MCI and AD-D fibroblasts was greater than that in CTRL fibroblasts treated with OA, indicating that AD-MCI and AD-D fibroblasts are more sensitive to OA treatment (Fig. S1a, b).

We also analyzed the mitochondrial membrane potential by flux cytometry using a TMRM probe. We reported an increase in the ΔΨm in AD-MCI and AD-D fibroblasts compared with that in control fibroblasts, which reached significance only in AD-MCI fibroblasts (Fig. 3b). Although we did not observe significant correlations between TMRM or MitoSOX intensities and clinical data (CDR-SOB, MMSE) (Fig. S1c–d and f–h), we detected a positive and significant correlation between TMRM intensity and the β-amyloid plaque burden (PiB-GCI) (Fig. S1e).

To characterize the metabolic constants in our cohort of fibroblasts, we measured the oxygen consumption rate (OCR) and the extracellular acidification rate (ECAR) using Seahorse technology and a set of mitochondrial respiration modulators (oligomycin, FCCP, and rotenone/antimycin A) (Fig. 3c, k). This protocol allowed us to determine basal respiration (Fig. 3d), maximal respiration (Fig. 3e), spare respiratory capacity (Fig. 3f), oxygen consumption associated with ATP synthesis (Fig. 3g), and oxygen consumption associated with proton leak (Fig. 3h). We reported a significant increase in the basal respiratory rate and proton leak in AD-D fibroblasts compared to those in AD-MCI and CTRL fibroblasts (Fig. 3d, h). We also revealed an increase in the maximal respiratory rate in both AD-MCI (significant) and AD-D (almost significant) fibroblasts compared with that in CTRL fibroblasts (Fig. 3e). Moreover, we noticed an increasing trend in the spare respiratory capacity, corresponding to the difference between the basal and maximal respiration only in AD-MCI fibroblasts compared to that in CTRL and AD-D fibroblasts (Fig. 3f). Compared with CTRL-treated cells, ATP-linked respiration in AD-D-treated cells also tended to increase but did not reach statistical significance (Fig. 3g). The ECAR was also greater in AD-D fibroblasts than in CTRL fibroblasts at both baseline and under stress conditions (i.e., after the addition of oligomycin and FCCP) (Fig. 3i, j). These data unexpectedly indicate increased metabolic activity in AD-D fibroblasts and a trend toward increased metabolic activity in AD-MCI fibroblasts. However, we did not observe an increase in glycolysis- or OXPHOS-mediated ATP production (GlycoATP or MitoATP, respectively) in AD-D fibroblasts (Fig. 3k). Interestingly, correlation analyses revealed that proton leak significantly correlated with the CDR-SOB, MMSE and PiB-GCI (Fig. 3l–n). Taken together, these results support the occurrence of mitochondrial dysfunctions in AD-D fibroblasts and noticeable changes in AD-MCI fibroblasts compared to those in CTRL fibroblasts. These data also revealed high metabolic activity in AD-D fibroblasts, likely impacting mitochondrial fitness, as manifested by enhanced mitochondrial potential and proton leak activity, the latter of which is associated with the cognitive hallmarks of AD.

### There are defects in mitophagy and autophagy in AD-MCI and AD-D fibroblasts

**Fig. 4:**
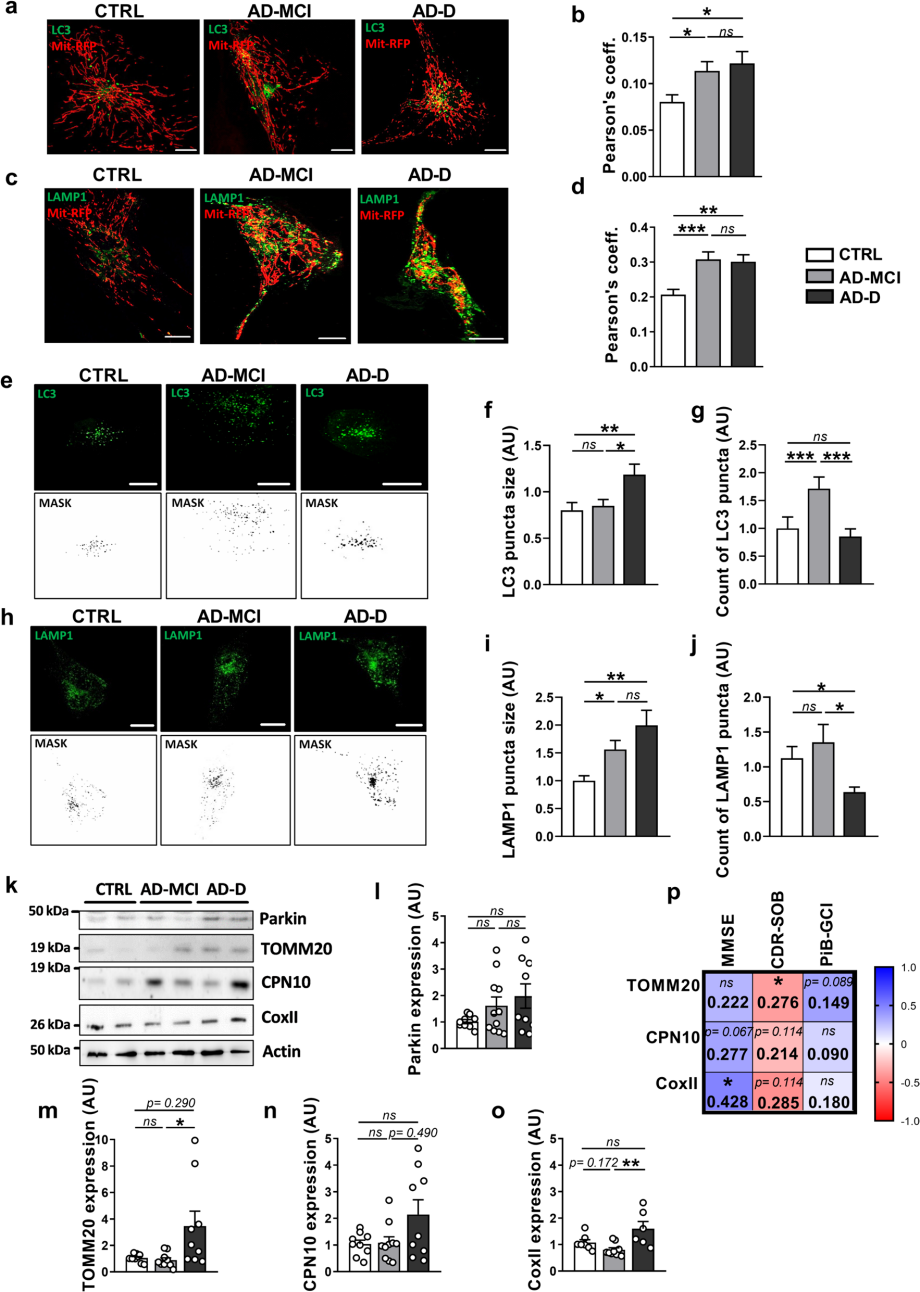
Mitophagosomes, mitolysosomes, autophagosomes and lysosomes as well as mitochondrial content analyses reveal defective mitophagy and autophagy processes in AD-D fibroblasts.** a**, **c** Representative images of Mit-RFP (red), LC3-GFP (**a**) or LAMP1-GFP (**b**) (green) signals in CTRL, AD-MCI and AD-D fibroblasts. The merged signal (yellow) reflects the colocalization of the red and green signals. Scale bars = 2 μm. **b**, **d** Quantitative graphs of the colocalization (Pearson’s coefficient) of Mit-RFP with LC3-GFP from CTRL (5 individuals, n = 42 cells), AD-MCI (6 patients, n = 49 cells), and AD-D (5 patients, n = 33 cells) (**b**) or of Mit-RFP with LAMP1-GFP from CTRL (5 individuals, n = 41 cells), AD-MCI (5 patients, n = 40 cells), and AD-D (5 patients, n = 40 cells) (**d**). Means ± SEMs *p < 0.05; **p < 0.01; ***p < 0.001; ns: not significant. **e**–**j** Representative images and masks of LC3-GFP (**e**) and LAMP1-GFP (**h**) signals in CTRL, AD-MCI and AD-D fibroblasts. Scale bar = 2 µm. **f**, **i** Quantitative graph of the number ± SEM of LC3-GFP puncta (**f**) and LAMP1 puncta (**i**). **g**, **j** Quantitative graph of the size ± SEM of LC3-GFP puncta (**g**) and LAMP1 puncta (**j**). The data were obtained from the CTRL group (5 individuals, n = 41 cells), AD-MCI group (6 patients, n = 49 cells) and AD-D group (5 patients, n = 40 cells). *p < 0.05; **p < 0.01; ***p < 0.001; ns: not significant. **K–n** SDS‒PAGE (k) and quantitative graphs of Parkin (**l**), TOMM20 (**m**), CPN10 (**n**), and CoxII (**o**) expression levels ± SEM obtained in mitochondria-enriched fractions of CTRL (n = 9), AD-MCI (n = 11) and AD-D (n = 9) fibroblasts. The data were normalized to those of CTRL fibroblasts. *p < 0.05; **p < 0.01; ns: not significant. **p** Heatmap of the correlation matrix computed linear regression (R^2^) between TOMM20, CPN10 and CoxII expression levels and the clinical Dementia Rating (Sum of Boxes) (CDR-SOB), the Mini Mental State Examination (MMSE) and the β-amyloid plaque burden assessed using the ^11^C‐labelled Pittsburgh compound B (Global Cortical Index) (PiB-GCI) through positron emission tomography (PET-scan) at patient inclusion. A color scale of 1 corresponds to the maximum positive correlation value (blue), and a scale of − 1 corresponds to the maximum negative correlation value (red). *p < 0.05 for every pair of data sets

We then studied mitophagy, the specific process involved in the degradation of dysfunctional mitochondria. We first showed an increase in the colocalization of the LC3-GFP probe with the Mit-RFP probe, demonstrating that the number of mitophagosomes is significantly greater in AD-MCI and AD-D fibroblasts than in CTRL fibroblasts (Fig. 4a, b). We also found a significant increase in the localization of the LAMP1-GFP probe with the Mit-RFP probe in both AD-MCI and AD-D fibroblasts compared to that in CTRL fibroblasts, reflecting an increase in the number of mitolysosomes (Fig. 4c, d). Together, these results suggest enhanced mitophagy in AD-MCI and AD-D fibroblasts. In addition, we reported that LC3 puncta size was significantly increased in AD-D fibroblasts than in those in the CTRL and AD-MCI groups (Fig. 4e, f) and that the number of LC3 puncta was significantly greater in AD-MCI fibroblasts than in those in the AD-D group. These results may suggest differences in lysosomal degradation between AD-MCI and AD-D fibroblasts. Accordingly, we showed a significant increase in the size of LAMP1 puncta in AD-MCI and AD-D fibroblasts compared to that in CTRL fibroblasts (Fig. 4h, i) and a significant decrease in the number of LAMP1 puncta in AD-D fibroblasts (Fig. 4h, j). These results (Fig. 4e–j) further support swollen and potentially dysfunctional lysosomes in AD-D fibroblasts and a mitigated phenotype in AD-MCI fibroblasts.

SDS‒PAGE analysis of the mitochondria-enriched fraction revealed an increasing trend (not significant) in parkin and CPN10 in AD-D fibroblasts (Fig. 4k, l and n) and a significant increase in TOMM20 and CoxII mitochondrial proteins in AD-D fibroblasts (Fig. 4k, m and o), which correlated with MMSE (i.e., CoxII) and CDR-SOB (i.e., TOMM20) scores but not with the PiB-GCI score (Fig. 4p). Taken together, these data suggest the recruitment of dysfunctional mitochondria to phagolysosomes in AD-MCI and AD-D fibroblasts (Fig. 4a–d) and the defective degradation of dysfunctional mitochondria by lysosomes in AD-D fibroblasts (Fig. 4e–o).

### Lysosomal degradation is defective in AD-D fibroblasts and correlates with cognitive deficits

**Fig. 5:**
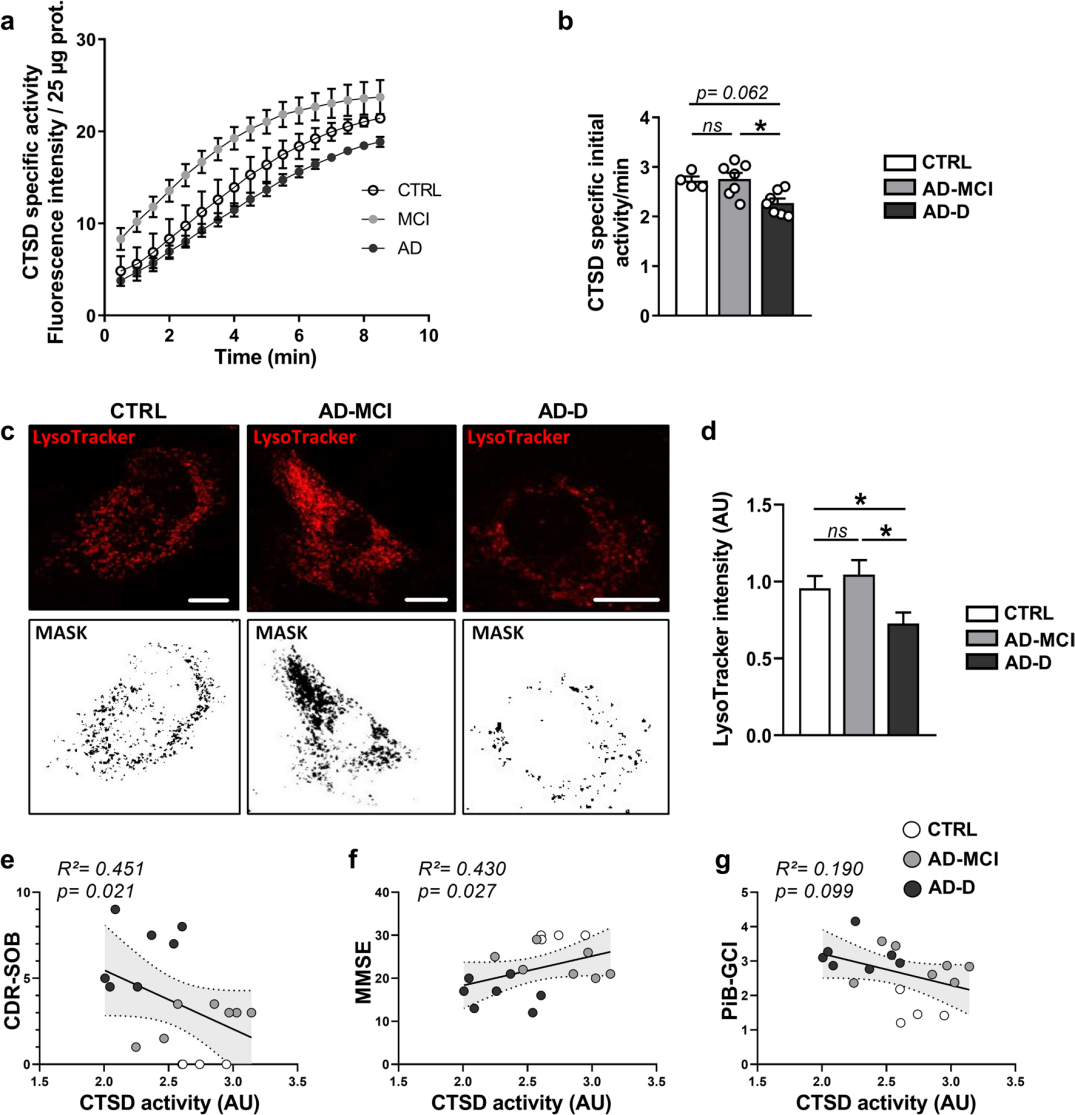
Lysosomal pH and cathepsin D activity analyses demonstrate defects in lysosomal degradation in AD-D fibroblasts. **a** Representative images and masks of LysoTracker signals in CTRL, AD-MCI and AD-D fibroblasts. Scale bar = 2 µm **b** Quantitative graph of LysoTracker mean intensity ± SEM obtained from CTRL (4 individuals, n = 142 cells), AD-MCI (6 patients, n = 211 cells) and AD-D (3 patients, n = 131 cells) fibroblasts. *p < 0.05; ns: not significant. **c**, **d** Cathepsin D (CTSD) mean specific activity ± SEM (**c**) and quantification of the initial specific activity ± SEM (**d**) obtained from the CTRL (n = 4), AD-MCI (n = 7) and AD-D (n = 7) groups. *p < 0.05; ns: not significant. **e**–**g** Correlation plots between CTSD activity and the Clinical Dementia Rating (Sum of Boxes) (CDR-SOB) (**e**), Mini-Mental State Examination (MMSE) score of patients (**f**) and the β-amyloid plaque burden assessed using the ^11^C‐labelled Pittsburgh compound B (Global Cortical Index) (PiB-GCI) through positron emission tomography (PET-scan) (**g**) at patient inclusion, including CTRL (white dots), AD-MCI (gray dots) and AD-D (black dots). Linear regression was used to determine *the* goodness of fit (R^2^) value

To assess whether the above-described LAMP1 alterations (enhanced size and reduced number) in AD-D fibroblasts are linked to lysosomal defects, we measured lysosomal activity by using complementary approaches. We first measured cathepsin D (CTSD) lysosomal enzyme activity (Fig. 5a, b) and reported a significant decrease in CTSD-specific initial activity in AD-D fibroblasts compared with that in CTRL and AD-MCI fibroblasts (Fig. 5a, b). Since the obtained results were homogeneous within each group, we used a LysoTracker Red probe to stain functional acidic lysosomes (pH 4) in a set of fibroblasts from the cohort and revealed a significant decrease in LysoTracker intensity in AD-D fibroblasts compared with that in CTRL and AD-MCI fibroblasts (Fig. 5c, d).

Together, these results consistently demonstrate a defect in lysosomes (low CTSD activity and defective acidification) in AD-D fibroblasts. Interestingly, we revealed a significant negative correlation between CTSD activity and the CDR-SOB score as well as a positive correlation with the MMSE score (Fig. 5e, f), while CTSD activity did not correlate with the PiB-GCI score (Fig. 5g). Together, these data (Figs. 4, 5) fully demonstrate that defective autophagy and mitophagy processes in AD fibroblasts are linked to a failure of lysosomal degradation and are correlated with cognitive deficits (CDR-SOB as well as MMSE).

### APP and APP-CTFs accumulation in the mitochondria of AD-D fibroblasts is correlated with cognitive deficits, mitochondrial protein load and reduced cathepsin D activity

**Fig. 6:**
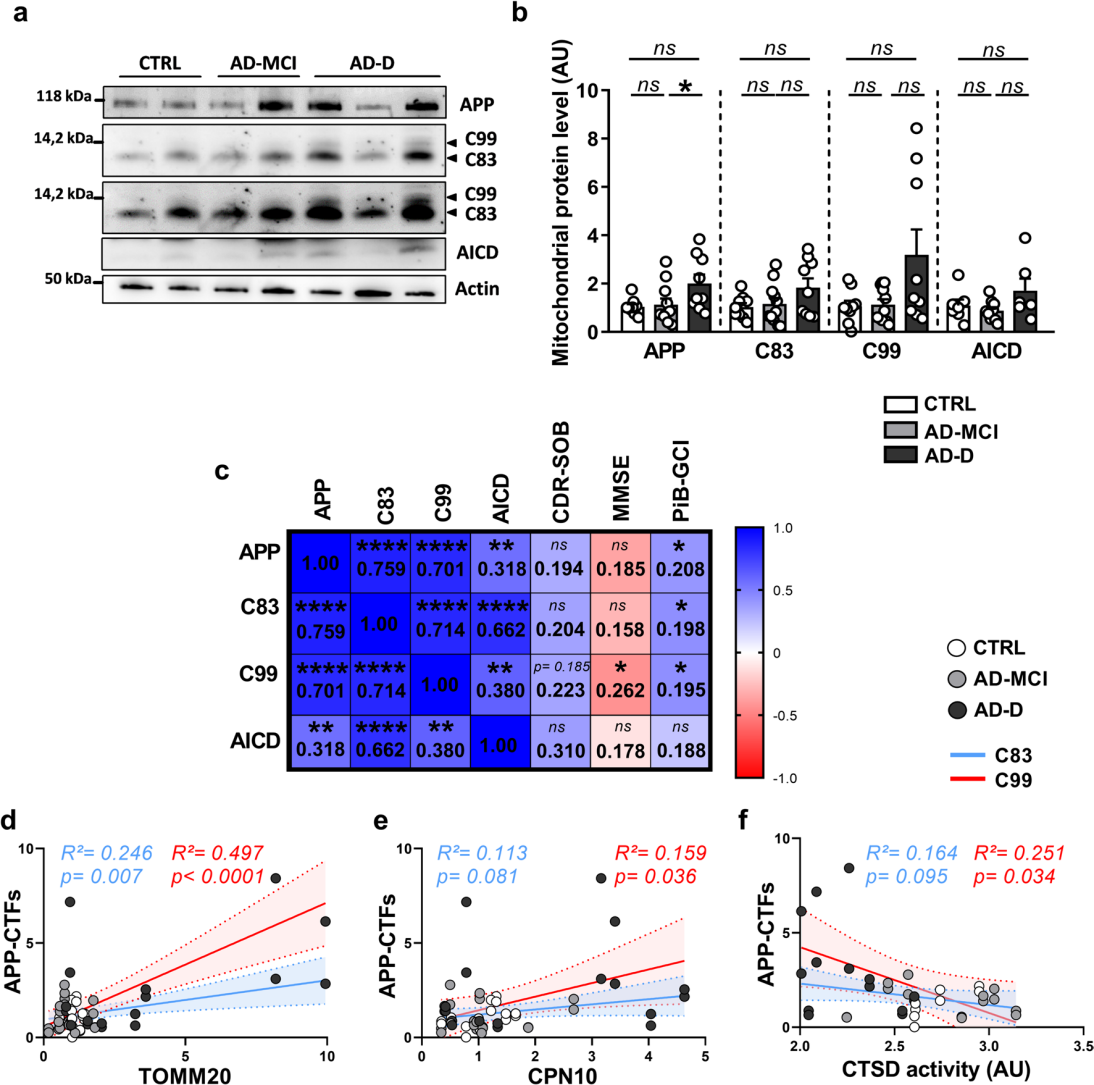
APP and APP-CTFs accumulation in the mitochondria of AD-D fibroblasts is correlated with cognitive impairments, reduced mitochondrial protein load and reduced CTSD activity. **a**, **b** Representative WB (**a**) and quantitative graphs of the expression levels ± SEM (**b**) of full-length APP (APP Total) and APP-CTFs (C99, C83 and AICD) obtained from mitochondria-enriched fractions of CTRL (n = 9), AD-MCI (n = 11) and AD-D (n = 9) fibroblasts. The data were normalized to those of CTRL fibroblasts. *p < 0.05; ns: not significant. **c** Heatmap of the correlation matrix computed via linear regression (R^2^) between the protein levels of full-length APP, C83, C99 and AICD fragments and the clinical Dementia Rating (Sum of Boxes) (CDR-SOB), Mini Mental State Examination (MMSE) and β-amyloid plaque burden assessed using the ^11^C‐labelled Pittsburgh compound B (Global Cortical Index) (PiB-GCI) through positron emission tomography (PET-scan) at patient inclusion. A color scale of 1 corresponds to the maximum positive correlation value (blue), and a scale of − 1 corresponds to the maximum negative correlation value (red). *p < 0.05; **p < 0.01; ****p < 0.0001; ns: not significant for the correlation of every pair of data sets. **e**–**f** Correlation plots between APP-CTFs and TOMM20 (**d**), CPN10 (**e**), or CTSD activity (**f**) in the CTRL (white dots), AD-MCI (gray dots) and AD-D (black dots) groups. Linear regression with C99 (red) or C83 (blue) was used to determine the *P* and goodness of fit (R^2^) values

Several studies have reported that full-length APP is localized [27, 28] and processed in mitochondria [29–31]. Accordingly, APP metabolites (C99, AICD and Aβ) accumulate in mitochondria-associated membranes [31–33]. Furthermore, our team and others recently reported that mitochondrial structure and functional defects, as well as mitophagy failure, are triggered by APP-CTFs accumulation within mitochondria [5, 34]. Hence, we sought to analyze the expression levels of full-length APP and APP-CTFs (C99, C83 and AICD) in the mitochondrial fraction of fibroblasts from CTRL, AD-MCI and AD-D patients. SDS‒PAGE analyses revealed the accumulation of full-length APP and a trend toward an increase in the levels of C99 in AD-D fibroblasts compared to those in CTRL and AD-MCI fibroblasts (Fig. 6a, b). Interestingly, linear regression analyses revealed statistically significant positive correlations between the expression levels of full-length APP, C83 and C99 fragments and the Aβ load measured by the PiB-GCI (Fig. 6c). We also highlighted a negative correlation between the MMSE score and the expression of C99, but not APP or C83 (Fig. 6c). In addition, while the accumulation of APP-CTFs did not correlate with either mitochondrial structure (Fig. S2a, b) or mitochondrial function (Fig. S2c–e), interestingly, we revealed a positive correlation between the accumulation of APP-CTFs and the accumulation of the mitochondrial proteins TOMM20 (i.e., C99 and C83, with greater significance for C99) and CPN10 (i.e., C99) and a negative correlation between the accumulation of APP-CTFs and CTSD activity (i.e., C99) (Fig. 6d–f). Taken together, these data suggest that the accumulation of APP and APP-CTFs in the mitochondria of AD-D fibroblasts is closely related to cognitive deficits and lysosomal defects in AD-D patients.

## Discussion

Several clinical studies have suggested that fibroblasts obtained from AD patients harbor cellular dysfunctions observed in postmortem AD brains [20]. In this study, we investigated mitochondrial structure and function and examined autophagy and mitophagy processes in fibroblasts obtained from AD patients to identify potential correlations between changes in mitochondrial homeostasis and the clinical onset of AD.

First, we report an alteration in mitochondrial ultrastructure (Fig. 1) and a decrease in mitochondrial volume and average branch length in AD-D fibroblasts compared to those in AD-MCI and control fibroblasts (Fig. 2). These results are in line with previous studies conducted in distinct SAD-derived fibroblasts [12, 13, 35, 36]. In addition, other studies have shown that fibroblasts from FAD patients also exhibit a reduction in mitochondrial number [15, 37]. Together, these studies, in addition to revealing alterations in the integrity and morphology of the mitochondrial network commonly observed in SAD and FAD fibroblasts, agree well with the data reporting fragmented mitochondria in postmortem AD brain samples [38]. Mitochondrial phenotypic changes are dependent on the balance between mitochondrial fusion and fission processes, which are governed by Mitofusin 1 and 2 (MFN1, MFN2), which form homotypic and heterotypic interactions with the OPA1 protein to initiate mitochondrial fusion, and by the FIS1 protein, which interacts with dynamin-related protein 1 (DRP1) at mitochondrial fission sites [39]. Interestingly, Wang et al. [38] reported altered expression of mitochondrial fusion and fission proteins in cortical samples from AD patient brains, supporting enhanced mitochondrial fission. Similarly, Manczak et al. [40] showed that the mRNA and protein levels of FIS1 and DRP1 are increased in the frontal cortex of patients with early, definite, and severe AD. However, alterations in mitochondrial structure phenotypes in AD fibroblasts are not corroborated by consistent modulation of mitochondrial fission and fusion protein expression. Pérez et al. [12] reported increased MFN1 expression in SAD fibroblasts, suggesting a compensatory mechanism, as mitochondrial hyperfusion may protect mitochondria from excessive mitophagy during stress conditions. A recent study by Drabik et al. [24] demonstrated a decrease in the frequency of fusion-fission events in SAD fibroblasts, which was associated with a reduction in the expression of both fission and fusion proteins.

Mitochondrial morphology finely shapes organelle function and vice versa. Hence, both mitochondrial membrane depolarization and hyperpolarization are considered key features of mitochondrial damage [41, 42]. Furthermore, while the controlled production of mitROS participates in mitochondria-nucleus communication [43], excess mitROS production is deleterious because it leads to oxidative damage to mitochondrial and cellular proteins, lipids and DNA [44]. Although we did not observe a difference in MitROS levels between AD-MCI and AD-D fibroblasts, we detected an increase in mitROS levels under stress conditions (OA) in both AD-MCI and AD-D fibroblasts (Fig. S1a, b). This observation corroborates the results of other groups describing an increase in mitROS in SAD [12, 45–47] and FAD fibroblasts [48] and demonstrates that mitROS are commonly enhanced in SAD and FAD fibroblasts, likely occurring at early disease stages.

Our data revealed that while basal and maximal respiration are enhanced in AD-D fibroblasts, only maximal respiration is increased in AD-MCI fibroblasts. These results point to specific metabolic signatures in AD-D and AD-MCI fibroblasts, revealing, on the one hand, a greater metabolic capacity in AD-D fibroblasts and, on the other hand, a higher adaptive capacity of AD-MCI fibroblasts to stress conditions. Our results corroborate the early mitochondrial hypermetabolism reported in the hippocampus of two *App* knock-in mouse models of AD (*App*^*NL−F*^ and *App*^*NL−G−F*^) [49], as demonstrated in mitochondria isolated from the hippocampus of young mouse models of AD prior to disease development. Mitochondrial hypermetabolism in these models was linked to the overexpression of OXPHOS-related genes [49]. We also postulate that the increase in Δψmit observed in AD-D and AD-MCI fibroblasts is the consequence of enhanced mitochondrial activity in these cells. In a different scenario, other studies have shown that chronic complex I inhibition and concomitant low complex II, III and IV inactivity enhance the mitochondrial membrane potential [50]. Accordingly, impairment of mitochondrial complexes I and IV has been reported in AD [50]. Moreover, enhanced Δψmit could also be attributed to the reverse-mode action of the mitochondrial adenosine nucleotide translocase (ANT) combined with ATP hydrolysis by the complex V reverse mode [50, 51]. The implication of this last mechanism in AD is supported at least in part by the interaction of Aβ and Tau with ANT in AD models [52]. Interestingly, a study by Naia et al. [49] showed that mitochondrial activity shifts from a state of hypermetabolism to a state of hypometabolism during AD development. This interesting issue merits further investigation in a longitudinal study of peripheral cells from AD patients. However, it is important to mention that in the current study, we used primary fibroblasts with limited passages and biological material, which impeded us from investigating the molecular mechanisms underlying mitochondrial hyperactivity and mitochondrial membrane hyperpolarization in AD-D and AD-MCI fibroblasts.

Moreover, we also showed that proton leak is enhanced in AD-D fibroblasts. Under physiological conditions, proton leak occurs to reduce the extent of mitochondrial hyperpolarization to maintain mitochondrial homeostasis and physiological ROS levels. In fact, it is known that increased ROS generation activates mechanisms that promote proton leak; and that enhanced proton leak in turn reduces ROS production, limiting further damage to mitochondrial function [53]. Therefore, proton leak in AD-D fibroblasts is likely driven by enhanced mitochondrial respiration and elevated Δψmit. In addition, we assume that proton leak may act as a compensatory mechanism to alleviate the increase in mitROS. In fact, the mitROS level was unchanged in AD-D under basal conditions (Fig. 3a) and was enhanced when mitochondrial coupled respiration was blocked by OA treatment (Fig. S1a, b). Interestingly, similar to our observations in AD-D cells, enhanced proton leak has been described under disease conditions and in aging [53, 54]. Mechanistically, active proton leak occurs through ANT and uncoupling proteins (UCPs), the expression and activity of which could also be altered in AD and contribute to mitochondrial demise. Moreover, the access to primary fibroblasts allowing only limited passages did not allow us to investigate the genuine mechanisms underlying mitochondrial proton leak in AD-D fibroblasts.

Mitochondrial structure alteration (fragmentation) and increased mitROS production are major features linked to mitophagy [55]. Our data indicate enhanced mitophagosome and mitolysosome formation in AD-D versus CTRL fibroblasts (Fig. 4a–d), supporting mitophagy initiation in AD-D fibroblasts. However, we observed an enlargement of autophagosomes (LC3 dot size) and lysosomes (LAMP1 dot size), which is indicative of defective degradation processes (Fig. 4e–j). Previous studies have reported an enlargement of endosomes in both PBMCs and fibroblasts of the IMABio3 cohort [17, 20]. Overall, these results demonstrate a global alteration of the endolysosomal compartments in SAD and FAD fibroblasts [56–58]. AD-D fibroblasts exhibited a significant increase in the levels of mitochondrial proteins (i.e., TOMM20 and CoxII) (Fig. 4k–n). This effect seems to be linked to mitophagy defects. Accordingly, an increase in TOMM20 protein levels [56] and a mitophagy failure molecular signature were also described in SAD brains [5]. Increased mitochondrial proteins in AD-D fibroblasts could also be linked to enhanced mitochondrial biogenesis. However, previous studies have reported that the expression of genes involved in mitochondrial biogenesis, such as PPAR-α, PPAR-γ coactivator-1 alpha (PGC-1α), NRF1, NRF2, and TFAM, is significantly decreased in AD [59–62].

The enlargement of lysosomes in AD-D fibroblasts supports a defect in their activity that was linked to decreased CTSD protease activity and to reduced acidification of lysosomes (Fig. 5a–d). We also revealed a correlation between CTSD activity and cognitive decline (Fig. 5e, g). Interestingly, a genome-wide association study revealed that the *CTSD* and cathepsin B (*CTSB*) genes are potential genetic risk factors for AD [63, 64]. Together, these data suggest that the failure of mitophagy/autophagy in AD-D fibroblasts is linked to lysosomal defects and may constitute a reliable indicator of AD progression in peripheral cells.

APP-CTFs accumulate in the brains of AD patients [5, 65] and may serve as potential biomarkers for AD [66]. We report herein a trend toward increases in APP and APP-CTFs (C83, C99 and AICD) protein levels in the mitochondrial fractions of AD fibroblasts that correlate with AD-related neuropsychological scores (Fig. 6). Since we did not observe a change in the expression of APP or APP-CTFs (C83, C99 and AICD) in the total extracts of AD-MCI and AD-D fibroblasts compared with those of the controls (data not shown), we concluded that the increase in APP in the mitochondrial fraction was not linked to increased APP expression but to its accumulation in mitochondria. Our study and others have demonstrated that APP-CTFs accumulation triggers mitochondrial structure and functional deficits as well as mitophagy failure [5, 31, 34]. In parallel, altered mitochondrial fitness and homeostasis could also affect APP processing as well as Aβ production, leading to a vicious cycle in the pathophysiological process of AD [67].

Finally, we showed that APP-CTFs accumulation was positively correlated with mitochondrial protein levels but not with alterations in mitochondrial structure or function. These data led us to assume that peripheral mitochondrial alterations observed in AD patients are not exclusively linked to APP-CTFs accumulation. Indeed, many mitochondrial alterations accompanying the pathophysiological development of SAD have been suggested to be driven by genetic risk factors [68]. In particular, several mitochondrial genes [63], including *TOMM40* (a component of the cation-selective translocation pore of the outer membrane translocase (TOM) complex), which is located within the *APOE* genetic risk factor locus, have been shown to be associated with SAD [69]. Furthermore, APP, Aβ peptides and likely APP-CTFs interact and/or are imported into mitochondria through the TOM complex [27, 70]. Polymorphisms in *the TFAM* gene, encoding the mitochondrial transcription factor A (TFAM) protein, were also associated with late-onset AD risk [63, 71, 72]. TFAM expression is reduced in both MCI and AD PBMCs and correlates with AD severity and mtDNA content [73]. Polymorphisms in the *COX7C* gene, encoding the COX7C protein, a component of OXPHOS complex IV, were recently described in the IMABio3 cohort [20]. Moreover, COX7C is expressed at lower levels in the blood of individuals with MCI/AD than in that of age-matched controls [74]. Finally, a new variant of a mitochondrial microprotein called SHMOOSE (*SHMOOSE. D47N*), is associated with AD risk and rapid cognitive decline and is correlated with reduced cortical volume [75]. SHMOOSE increases the oxygen consumption rate, boosts the mitochondrial spare respiratory capacity and binds the mitofilin protein in the mitochondrial inner membrane, which is known to regulate cristae junctions [75]. Interestingly, SHMOOSE-related functions are also altered in IMABio3 AD fibroblasts.

To our knowledge, this is the first study reporting mitochondrial ultrastructure, morphology, functional alterations and mitophagy dysfunctions in fibroblasts obtained from a clinically characterized SAD cohort including AD-MCI and AD-D patients. This study revealed that the observed mitochondrial alterations mostly recapitulated those reported in AD brain samples. Accurate AD diagnosis is still needed; thus, it is essential to uncover new peripheral biomarkers for the identification of early changes in peripheral cells/fluids in AD patients. Our study provides evidence that mitochondrial alterations and defects in lysosomal degradation in peripheral tissues are potential biomarkers of the progression of AD. For the first time, we report the accumulation of APP-CTFs in the mitochondria of AD fibroblasts. Therefore, peripheral APP-CTFs levels might also be considered an additional pathophysiological marker of AD.

Overall, this study provides insight into the molecular mechanisms underlying mitochondrial dysfunction in peripheral AD cells.

### Supplementary Information

Supplementary material 1

## Data Availability

Not applicable to this article as no datasets were generated or analysed during the current study.
