# Supplementary material for: Mitochondrial alterations in fibroblasts from sporadic Alzheimer's disease (AD) patients correlate with AD-related clinical hallmarks
Source: Acta Neuropathol Commun. 2024 Jun 8;12:90. doi: 10.1186/s40478-024-01807-x (PMC11161956; doi:10.1186/s40478-024-01807-x)
Supplement: Supplementary file 1 — Supplementary material 1 [file 40478_2024_1807_MOESM1_ESM.pdf]

Supplementary Material and Methods

Mitochondrial superoxide measurements under stress condition by confocal microscopy

As for Flux cytometry, cells were loaded with MitoSOX at 5 µM in DMEM for 30 min, at 37 °C and rinsed with KRB buffer (135mM NaCl, 5mM KCl, 1mM MgSO4, 0.4 mM K2HPO4, 20 mM HEPES, 0.05 mM CaCl2, 1g/L Glucose, pH 7.4). We analyzed MitoSOX signal by confocal microscopy at the cellular level under stress condition (oligomycin (10µM) + Antimycin A (1 µM) overnight). Confocal images were acquired by live imaging using Zeiss LSM 780 with 63X objective. MitoSOX fluorescence median intensities were analyzed on Z projections using Fiji software.

Fig. S1. Mitochondrial superoxide measurements under stress condition and correlation analyses between the mitochondrial function and the patients' clinical data. **a** Representative confocal microscopy images of fibroblasts treated with Oligomycin and Antimycin A (+OA) and stained with MitoSox probe. Scale bar= 10µm. **b** Quantitative graph of Mitosox mean intensity ± SEM obtained by confocal microscopy analyses from CTRL (4 individuals, n= 100 cells), AD-MCI (4 patients, n= 92 cells) and AD-D (4 patients, n= 122 cells). **c-h** Correlation plots between TMRM (c-e) and MitoSox (f-h) and the CDR-SOB, MMSE, and PiB-GCI at patient's inclusion including CTRL (white dots), AD-MCI (grey dots) and AD-D (black dots). The linear regression was used to determine *P* and goodness of fit (R^2) values.

Fig. S2. Correlation analyses between the accumulation of APP-CTFs and mitochondria structure and function. **a-e** Correlation plots between APP-CTFs and mitochondria classes I (a) and IV (b), TMRM mean intensity (c), MitoSox mean intensity (d), and proton leak (e) including CTRL (white dots), AD-MCI (grey dots) and AD-D (black dots). The linear regression with C83 (blue) or C99 (red) were used to determine *P* and goodness of fit (R^2) values.

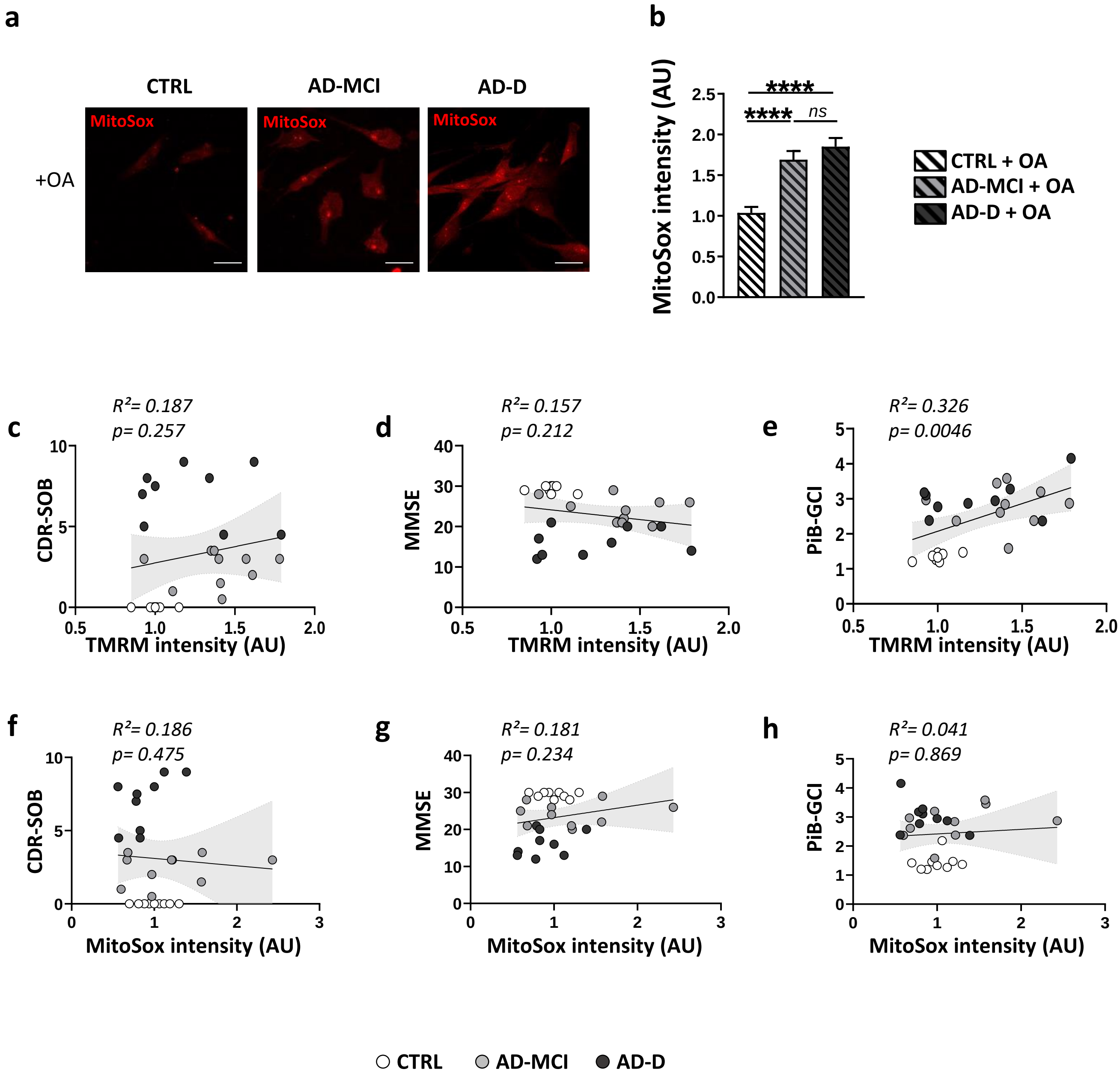

Eysert et al., Fig. S1

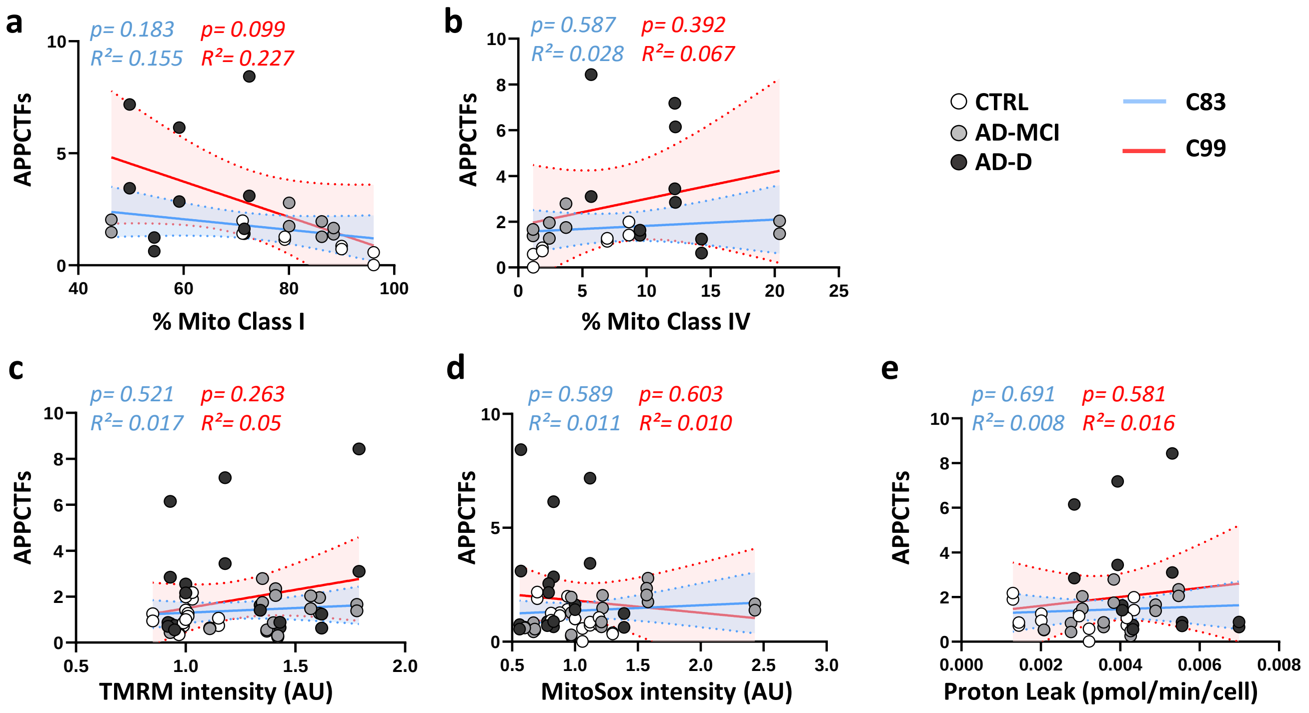

Eysert et al., Fig. S2
